# Prostate cancer awareness, case-finding, and early diagnosis: Interviews with undiagnosed men in Australia

**DOI:** 10.1371/journal.pone.0211539

**Published:** 2019-03-07

**Authors:** Ashwini Kannan, Maggie Kirkman, Rasa Ruseckaite, Sue M. Evans

**Affiliations:** 1 Clinical Registry Unit, Department of Epidemiology and Preventive Medicine, Monash University, Melbourne, Australia; 2 School of Public Health and Preventive Medicine, Monash University, Melbourne, Australia; King's College London, UNITED KINGDOM

## Abstract

Previous research in Victoria, Australia, found differences in prostate cancer outcomes in regional and metropolitan areas. This investigation of undiagnosed men in regional areas and a metropolitan area of South Australia sought their perspectives on prostate cancer. Our aim was to learn whether men who had not been diagnosed could shed light on why men outside metropolitan areas tended to have poorer outcomes than metropolitan men. Our goal was to build on evidence contributing to improving outcomes in prostate cancer care. Semi-structured interviews were designed to elicit explanation and meaning. 15 men (10 metropolitan, 5 regional) not diagnosed with prostate cancer were recruited through widely-distributed flyers in medical and community settings. Interviews were recorded and transcribed; transcripts were analysed thematically. Five main themes were identified, four of which were prompted by the questions: *Addressing prostate health*, *Experiences with and expectations of GP*s, *Differences in care between regional and metropolitan areas*, and *Achieving early diagnosis*. The fifth theme arose spontaneously: *Australian masculinity*. Men identified as problematic the limited availability of GPs in regional areas, the lack of consistency in approaches to prostate cancer detection, and men’s reluctance to seek medical care. Community-level strategies appear to be valued to encourage men to address prostate health. Maintaining and extending a systemic approach to prostate care may improve outcomes for men in Australia.

## Introduction

In 2011, the Victorian Cancer Registry (VCR) reported that men diagnosed with prostate cancer in one regional integrated cancer service (RICS) in Victoria, Australia, had a 7% lower 5-year age-standardised survival rate than in a metropolitan region (93% vs 86%, p<0.001) [1]. In order to investigate the identified disparity between the RICS and other regions of Victoria, data from men diagnosed with prostate cancer retrieved from the VCR and the Prostate Cancer Outcome Registry-Victoria (PCOR-Vic) were analysed [2]. Clinical, sociodemographic and quality of life differences between men diagnosed with prostate cancer in the RICS were compared with other Victorian regions. It was found that men in the RICS were more likely to be diagnosed with more advanced prostate cancer and to have poorer outcomes than metropolitan men. Further qualitative inquiry of men with and without prostate cancer and of GPs identified challenges associated with living in regional areas, including limited access to health services, GPs, and specialists; limited understanding of prostate cancer; and regional men’s reluctance to consult their GPs (who were often well known in the community) about sensitive matters such as prostate cancer, with concomitant digital rectal examinations [3]. Differences between metropolitan and rural men’s health are not confined to Australia, a topic we have discussed elsewhere [3]. GPs requested more consistent guidelines, noting, for example, that the Royal Australian College of General Practitioners (RACGP) discouraged screening unless men requested it and the National Health and Medical Research Council recommended screening for men at average and high risk [4, 5].

The research reported here was undertaken to extend knowledge gained from men in Victoria to another state, South Australia, where a substantial proportion of its population lives in regional and remote areas. Our aim was to understand the perspectives of men who had not been diagnosed with prostate cancer on its detection and management. Although we were particularly interested in how men assessed the role of place of residence in health outcomes, we did not set out to compare men according to group membership but to learn from the reflections of individual men. Our goal was to contribute evidence that could be used to improve men’s health, particularly prostate cancer care.

## Materials and methods

### Design

Qualitative methods are appropriate for seeking to understand personal perspectives and meaning [6]. Our approach to understanding was phenomenological. Because we were investigating particular components of a specific topic, semi-structured interviews were the most suitable means of gathering data [6].

### Settings, participants and recruitment

Men were eligible to participate if they had no history of prostate cancer and were aged from 40 to 80 years. Volunteers were sought through electronic notices posted to Monash University’s Facebook and Twitter accounts; emailed to shopping centres, community clubs, Rotary clubs, libraries, GP clinics, and Men’s Sheds [7] throughout metropolitan, regional, and rural South Australia; and posted through an account on a popular online selling and trading site (Gumtree). A $20 shopping voucher was offered as compensation.

Based on experience, 15 men were expected to provide informative data and were sought purposively to ensure a mix of metropolitan, regional, and rural residence (determined by Australian Bureau of Statistics [8] classification) and diversity in age and education. It has been found that metatheories can be identified after six interviews and that “data saturation” commonly occurs after twelve interviews [9]. The contentious subject of saturation was discussed but not formally employed because the research predominantly sought perspectives on a limited number of topics and was not theory-generating [10]. Men who contacted the research team to express interest were sent information about the research and a consent form. Volunteers consented either by returning signed consent forms or by recording their consent before the interview began.

### Data collection

A study-specific interview guide was developed based on previous research and the research aim (S1 Fig). The main topics covered were men’s experiences of prostate cancer, the role of the GP in prostate care, potential ways of increasing knowledge of prostate care, and men’s health in rural and remote areas. Questions were designed to encourage men to reflect on the topics and to pursue relevant matters of importance to them, rather than to limit them to researchers’ expectations. Interviews were conducted by telephone and audio recorded, with permission. After each interview, interviewers made summary notes, especially of aspects that might not be fully captured in transcription.

### Data analysis

Audio files of interviews were transcribed. When each man had been given a pseudonym and identifying details deleted or disguised, transcriptions were uploaded onto NVivo11 [11] to assist with data management. Thematic analysis employed deductive and inductive coding [12]. Transcripts were initially searched for themes by AK, MK and RR, prompted by the interview guide (deductive codes). They were re-read several times seeking unexpected themes (inductive codes). Themes identified in the transcripts were discussed within the research team (AK, MK, RR and SE) to establish any organising patterns and sub-themes. All transcripts were read again to ensure that the final thematic scheme was comprehensive and appropriate. The method and results are reported according to the COREQ statement (S1 Table).

### Reflexivity and credibility

All researchers are women, of diverse ages. AK was a student trained and supervised by the other authors, each of whom has a PhD and is engaged in epidemiological or qualitative research on prostate cancer. AK was present at or conducted all interviews, led or supported by MK (an expert in qualitative research on sensitive topics) for the first five; discussion and debriefing occurred after each remaining interview. Apart from communication to arrange times for interviews, there were no prior relationships with participants. The participant information sheet gave basic information about the research team. AK conducted initial data analysis, guided by the other researchers who also participated in each stage of analysis and writing. As is expected in qualitative investigation, the researchers sought to understand the topic from the perspective of the participants, frequently seeking clarification. To this end, being women and (in the case of AK) from another country meant that participants were encouraged to explain ideas such as Australian masculinity without assuming that the interviewer would share an understanding.

### Ethics

Approval to conduct this research was granted by Alfred Health (122/17) and Monash University Human Research Ethics Committee, Melbourne Australia (HREC/17/Alfred/33).

## Results

Fifteen men (10 from metropolitan Adelaide, 5 from regional South Australia) were recruited from May to July 2017. The characteristics of these men are shown in Table 1. Despite seeking recruitment from rural and remote areas, there were no volunteers. It was not possible to establish how many men saw the invitation to participate but did not volunteer; no volunteers dropped out of the research. Interviews lasted on average 22 minutes (range 10–27) and no repeat interviews were required. Metropolitan men learnt about the research from Gumtree [9] (7) and Rotary Clubs (3); regional men learnt from Men’s Sheds (3) and word-of-mouth (2). The men were aged 40–75 years (mean 53), with the regional men older on average (61) than the metropolitan men (49); their ages were reflected in employment status, with 7 metropolitan men and 2 regional men in paid employment. All but two of the metropolitan men and one of the regional men were in a relationship. All but two men (both metropolitan) identified as Anglo-Australian; four men who were born overseas had lived in South Australia for over 20 years. Apparent socio-economic status and employment were diverse.

**Table 1:** Characteristics of the 15 men without prostate cancer who participated in the interviews.

Characteristic	Metropolitan (N = 10)	Regional (N = 5)	Total (n)
**Recruitment source (n)**			
*Gumtree*	7	0	
*Rotary Clubs*	3	0	
*Men’s sheds*	0	3	
*Colleague contacts*	0	2	
**Currently in a relationship (n**)	8	4	12
**Working status (n)**			
*Retired/unemployed*	3	3	6
*In paid employment*	7	2	9
**Ethnicity (n)**			
*Caucasian*	8	5	13
*Other*	2	0	2
**Mean age (range)**	49 (40–69)	61 (50–75)	53 (40–75)

### Themes

Four themes arising from the topics addressed by the interview guide were identified: *Addressing prostate health*, *Experiences with and expectations of GP*s, *Differences in care between regional and metropolitan areas*, and *Achieving early diagnosis*. There was one unexpected theme: *Australian masculinity*. Each is discussed in turn.

### Addressing prostate health

Men described diverse awareness of prostate health, apparently associated with their experiences, expectations, education, and professional contacts. For example, some men said that they knew little about prostate cancer apart from what they had learnt from public health campaigns on television. Men whose family members or friends had been diagnosed, such as Ross (*69*, *metropolitan*) whose father had been diagnosed, tended to be more aware of both the physical and emotional impact as well as the need for preventive measures. Those without this experience made comments such as “*It’s obviously something that happens to people and that’s just part of life*” (*Patrick*, *50*, *Regional*). Six volunteers had been attracted to the research because they were professionally associated with men’s health or were part of an organisation advocating for men’s health. One man referred to his experiences using medical terminology:

*“I’ve had an issue with BPH in the past… not so much concerned about prostate cancer but just the cystoscopy and the TURP side-effects*.*” (George*, *59*, *Regional*)

This contrasts with other accounts of biopsy presented in lay terms. Simon *(61*, *Regional)*, for example, described it as *“a small procedure involving an instrument come up my rectum taking a small snip*.” Risk and symptoms were presented in similarly diverse ways. Most men who revealed some knowledge of prostate cancer perceived themselves to be at higher risk because of their age. Four of the men with less apparent knowledge suggested symptoms that would prompt them to seek medical attention: pain, bleeding, lumps in the scrotum, irregular bowel movements, tiredness, loss of appetite, and changed sexual function. Aaron (*53*, *Metropolitan*) said, *“I have examined myself*, *because aren’t you supposed to check for if you have lumps or things like that*?*”* These symptoms however, are usually associated with testicular cancer. In the absence of symptoms, men who were vague about prostate cancer reported not seeking prostate checks from a GP. Sam (*44*, *Metropolitan*), for example, said, *“I’ve considered myself pretty fit and healthy*. *…*. *I only go and see the doctor if I’m really*, *really sick”*. Two participants assessed risk based on family history, including one with a family member who was a health professional:

*“I have type II diabetes and, since we have a family history, most of our discussions revolve around present problems. Prostate cancer has never been an issue in the family, so the topic doesn’t come up.”- Drake*, *47, Metropolitan*

These men assessed their risk according to symptoms and problems without mentioning early diagnosis. It had appeared that the PSA test was often part of a general health check in men who visited the GP rather than an approach solely to address prostate health.

### Experiences with and expectations of GPs

Participants’ experiences with GPs varied among the group. Approximately half the men reported good relationships with their GPs whereas other men were distrustful of doctors and the healthcare system. Men generally assessed the competency of doctors through their expectations of the GP’s proactivity in discussing prostate health as well as their inclusiveness in the decision-making process.

### Case finding

Similar experiences were reported by men if they felt motivated to be pre-emptive about their health from the influence of their proactive GPs. Some participants who had greater knowledge and insights into prostate cancer, such as those that actively campaigned for greater awareness of prostate cancer, reported having a more forthright approach with their GPs whereby they initiated discussions regarding undergoing prostate checks. Two such men stated that their GPs were surprised that they had raised such an issue, with one man saying:

*“It doesn’t seem to be right at the top of their list that they’re looking out for”- Sean, 58*, *Metropolitan*

Regardless of the GPs’ reaction, these men went on to describe how they persisted with their GP to get themselves tested for prostate cancer.

Conversely, some of the participants expressed dissatisfaction with their GPs as they felt that their care was not ‘*personalised’ (Sonny*, *44*, *Simon*, *61 and George*, *59)*. One participant particularly voiced his exasperation with the public health system saying:

*“You go to a bulk billed centre, there are 7 doctors and they see you in the order you’ve come in…They give you a quick fix for the problem you’ve come for. It’s not a relationship*, *it is transactional.”–Sonny, 44, Metropolitan*

Overall, the interviews found that men’s experiences with GPs typically influenced their ensuing expectations. Interestingly, most men did not feel that having a consistent family GP was important with reasons for this being related to time, convenience and preference for diversity in opinions from different doctors.

### The role of a good GP

When asked about what makes a good GP, men had differing opinions. Several men identified a good GP as someone who was able to identify the cause of their problems within the one consultation and would then be proactive in referring his patients to a specialist. Others however, expected GPs to be more approachable and open to discussion and to give their patients the time of day, listen, coax information out of the patients and give options rather than authoritatively determining the course of action. This was clearly reflected by one participant who said,

*“[A good GP is someone] who is happy to talk about issues and can explain things sometimes when you're not quite sure; so not just say well I think you should do this, but give you the options and then maybe talk about it [further].”–George*, *59, Regional*

### Differences in regional and metropolitan areas

There were notable differences in care in regional areas, particularly due to the limited availability of doctors as reported by regional participants. Most participants felt that attitudes of regional men differed from metropolitan men, in that they were more stoic and of a lower socioeconomic status which was likely to prevent them from addressing the topic of prostate health.

### Access to care

Men recruited from the regional areas of South Australia recounted differences in their access to primary care. Regional GPs were reported to be more elusive, were mostly from non-Australian backgrounds and had higher turnover rates than in metropolitan clinics. One participant pinpointed specific differences in metropolitan and regional care by explaining that metropolitan men got the whole prostate screening process done in 10 days whereas,

*“Here, you might get an appointment for a couple of weeks to a month. If you're a public patient it's hopeless, [Doctors] do those tests and then you know you might have to wait another three months before you see them and if you go on the public waiting list it could be three to six months before anything happens.”–Barry*, *59, Regional*

Most men from metropolitan areas also perceived regional men to be at a disadvantage due to difficulties in accessing healthcare. As aforementioned, a few men from metropolitan areas also reported a lack of satisfaction in the care they received due to the perceived impersonalised approach to patients in the public health system.

Other issues associated with GPs in regional areas included difficulty in communication due to their foreign accents and relative inexperience with Australian culture. There was also the perception that the available GPs were less inclined to focus on preventative health and were more interested in only treating the health problems with which patients presented.

### Characteristics of regional men

Both metropolitan and regional participants were asked about their views on the attitudes of regional men towards prostate care as summarised in Table 2.

**Table 2:** Participants’ views of regional men’s awareness of and attitudes to prostate health.

	Views of metropolitan men on regional men	Views of regional men on regional men
**Positive views**	“They would be more aware of the issue because they are so remote”	“Regional men are more self–sufficient and independent. This leads them to pay attention to detail”
**Indifferent views**	“Aussie men are just Aussie men”	-
**Negative views**	“Lesser intelligence and more bogan[13]”	“High access barriers and increased waiting times to consult urologists and GPs”
	“More macho”	“Lower SE and more stoic”
	“High access barriers”	“Isolation”
	“They don’t embrace things as freely”	“Less electronic literacy”

Despite the different views amongst participants in general, the quotes in Table 2 displayed that there were no apparent disparities in the views of both groups.

Some participants felt that regional men had greater awareness of their health because it was well established that people in the region had fewer resources and the recurring discussion around it created more awareness. One participant reflected this view by voicing that,

*“In the rural area they would look more into it because they're a distance from it, whereas in the city you wouldn’t be looking so much because it would be around you all the time”–Aaron*, *53, Metropolitan*

Also, some participants felt that regional men were a lot more independent and self-sufficient which led them to paying greater attention to their well-being and hence more aware of their prostate health. Other participants felt that there were no significant differences between regional and metropolitan men in terms of their awareness to prostate health. One participant concurred with this view by saying that

*“I can’t say that people in rural areas are dumb*, *and I can’t say that people in metropolitan are smart*, *it really depends on the individual*.*”–Sonny*, *44*, *Metropolitan*

Most other participants however, reported that most regional men had a different set of social determinants compared to metropolitan men. They determined regional men to be of a lower socioeconomic status and of lesser educational attainment thus reflecting poorer attitudes towards addressing prostate health and overall understanding of prostate cancer.

### Achieving early diagnosis

Men felt that early diagnosis was achievable through government support, GP’s proactivity and self-awareness. Creating awareness was determined to be most effective when men were actively involved in receiving information.

### Creating awareness

In determining what men felt was most effective in creating awareness, many raised the topic of active and passive advertising. Examples of passive advertisements included TV ads, social media and leaflets. However, some men felt that leaflets had little impact as they viewed reading information to be a highly passive process. One participant mentioned that,

*“The government sends out [a letter] when you turn 50, I’ve always just got that, but I haven’t even opened it”–Patrick*, *50, Regional*

Men who identified such methods to be passive, also described the most effective ways of discussing prostate health to be ‘active participation’. Sport-themed men’s health events were viewed as having a higher impact for getting men involved in a more relevant way. One participant reflected this view by suggesting,

*“Sport, if you wanted to promote prostate checks or prostate awareness, you would get some high profile sporting people, you would get them to go to sporting clubs as a guest speaker”–Ross*, *69, Metropolitan*

### Other methods of ensuring early diagnosis

GPs, government and individuals (themselves) were all cited by participants as having a significant role in ensuring early diagnosis. Some men felt that GPs acted as gatekeepers to discovering any issues relating to prostate health and that it was expected that GPs should initiate the discussion on prostate checks. One participant extended this view by saying that as men hardly ever go to the GP for general check-ups, GPs need to be opportunistic in bringing the topic up even if men come in for other issues. Other participants felt that the government had a role to play and distributing something akin to the bowel cancer screening kit currently received, may be useful to men. A small group of participants however, felt that awareness, ensuring an early diagnosis was up to each individual, and it was crucial to be proactive.

### Limitations associated with current awareness approaches

Some men identified limitations in current awareness approaches, particularly TV ads. One participant found that advertising was a futile effort; if it was not perceived of as a relevant and current problem that men resonated with, they were highly unlikely to respond to advertisements encouraging prostate checks. The participant described this using an analogy,

*“When you're keen on something you notice things, otherwise no matter how many times people tell you, you won't take cognisance of what is being said.”–Sonny*, *44, Metropolitan*

One of the participants who had more professional insights into health, felt that giving too much information from various sources to men was disadvantageous as it could lead to men over-analysing pros and cons of prostate cancer testing and might create confusion. This participant further explained that men’s health events should encourage men to speak to their GPs, rather than disseminating bits of information from different sources for men to interpret themselves.

### Australian masculinity

Participants’ stance in relation to the discourse on masculinity varied. While some men identified with the views discussed, other men showed resistance to it when interpreting the masculine perspectives and attitudes of other men.

### Men who identified with traditional views

Many participants admitted that speaking about prostate cancer amongst their peers or family was not a common experience. This was reportedly due to the stigma attached to prostate cancer and its association with the DRE. One participant voiced his discomfort around speaking about the topic by saying,

*“If you didn’t know what the test involved you'd probably [have] some friend joking about it saying you’ve got to bend over and someone’s going to insert something into your anus.”–Vlad, 40*, *Metropolitan*

Also, among most participants, there was a consensus on how an ‘average Australian male’ would behave. It was felt that most Australian men tried to brush their health concerns aside and it was especially unusual to discuss reproductive-related health matters with their peers. One participant even mentioned that,

*“Men don’t really consider being sensitive, and have a higher sort of threshold for pain compared to women.”–Ashton, 42*, *Metropolitan*

However, this view of hegemonic masculinity was opposed by another participant who said,

*“It’s essential to [talk] and not really great drama [because] when we look at the ladies in our life they’ve had to endure much worse.”–Simon*, *61, Regional*

Some men who positioned themselves outside of the discourse felt that men were more likely to be reticent about raising the topic of prostate cancer due to denial of, rather than embarrassment about the issue. The threat of diagnosis and position of vulnerability prevented men from addressing their prostate health. This was reflected by a participant who voiced,

*“It makes them feel weak or they're not in control basically when they’ve got to [talk].”–Barry*, *59, Regional*

### Men who resisted the stereotype

Men appeared to be more resistant to masculine norms when they had previous experiences of family or friends being diagnosed. One participant who was affected by a family history of prostate cancer said that,

*“I make it loud and clear to all my male friends around the same age that I get my prostate checked all the time, and do they get theirs checked, and generally the answer was no, and I said well get off your arse and get it done. Because prevention is better than medication, and if you can get early stage diagnosis, you may not require as much medication, or any.”–Ross, 69*, *Metropolitan*

The group of men with professional insights to health also appeared to resist the discourse and were especially passionate on having an open discussion on prostate health and screening. Most of these men were part of Rotary clubs and men’s sheds that held regular sessions educating men on various health issues and screening programmes. Participants from these groups felt that the environment of their clubs created a safe space for men to discuss sensitive issues such as prostate health. One participant highlighted the importance of having such an environment for men to discuss health related issues by sharing that,

*“[The] doctors always explain it well, but coming from someone of your own ilk who’s not a doctor is probably just as important.”–Simon, 61*, *Regional*

## Discussion

The main findings of the paper were that men had a low perceived risk of being diagnosed with prostate cancer particularly due to the absence of symptoms; heterogeneity in men’s understanding of prostate cancer in all aspects including anatomy, symptoms and current trends in Australia; identifying with or resisting the discourse of masculinity influenced men’s approach to addressing prostate health; and men generally were not proactive in monitoring their prostate health unless they had family or friends with a history of being diagnosed with prostate cancer, or they had professional insights to health.

Explanations based on allusions to “Australian masculinity”, whether or not they reflected the participants’ own perspectives, are consistent with results of other research that found the masculine stereotype to be one of stoic, silent endurance, in which help-seeking is unmanly [14]. This hegemonic masculinity has a long history and is not confined to Australia [15, 16]; vigilance and action are necessary to counteract its effects.

The findings of this study reinforce challenges identified in a study of perspectives of prostate cancer diagnosis and care of men with and without prostate cancer residing in regional Victoria [3]. The challenges include evidence that, because of men’s diverse experiences and environmental influences, a singular approach to improving awareness of prostate cancer is insufficient. Our results extend the earlier research by garnering views of men on creating awareness of prostate cancer in various appropriate ways.

Awareness of prostate cancer in Australia has been a subject of febrile debate, particularly in the past decade. A study conducted to analyse media coverage of prostate screening in Australia from 2003 to 2006 found that 388 print media articles and 42 televised news advertisements had discussed prostate cancer screening [17]. Awareness in this context was not necessarily in terms of advocating screening but rather to create a dialogue to address the contention. Despite this, it was reported that public discourse in this time period was skewed towards a supportive stance on screening as a result of the Australian media coverage of issues regarding prostate cancer [17]. The recommendations of men in this study regarding raising awareness in an interactive manner such as a sport-themed men’s health event [18] concurs with current initiatives run in Australia. Recent examples of awareness programmes include national and interstate events such as ‘Turning Australia Blue’, ‘Big Aussie Barbie’ and ‘It’s a Bloke Thing’, annually supported by the Prostate Cancer Foundation of Australia [19].

It might be informative to consider public health initiatives for prostate cancer prevention by reference to the Integrated Behaviour Model [20, 21] This model accounts not only for behaviour but for cognition and intent, including motives, knowledge, awareness, environmental constraints, and habit as ways of understanding and predicting behaviour. The importance of social influence is accepted. Continuing to refine and implement social awareness programmes, including those named above, has potential not only to challenge discourses of masculinity that discourage help-seeking behaviour such as prostate examinations, but also to encourage informed decision-making [22]. There is limited research evaluating the effectiveness of awareness programmes, resulting primarily from the heterogeneous information provided to men at different events. Appropriate, targeted programmes need to be built on further research and evaluation.

The importance of the relationship between the GP and the patient in terms of case-finding was also identified as a key finding of this study. According to guidelines of the RACGP, screening of prostate cancer in Australia is currently not recommended in asymptomatic or low-risk men [5]. The difference between screening and case-finding although subtle, is tangible. The main difference lies in ethical duty of the health professional. While both screening and case-finding refer to diagnosis, screening relies upon the ethical duty of the health professional to actively encourage prostate checks, whereas case-finding refers to GPs recommendations of a PSA test for men (in context of prostate cancer) based on patient profiling [23]. Our study has highlighted that case-finding is not expected to be prevalent as men tend to be highly unlikely to present themselves to GPs. Many men perceived they were at low risk of being diagnosed with prostate cancer because they were ‘fit and healthy’. This suggests that they would only present to a GP if they were symptomatic, generally an indication of more advanced disease [24]. Additionally, the shift to a model where Super Clinics are increasingly the norm and small individual practitioner clinics are disappearing may affect case finding practices, although the impact of this remains unclear. Diversification of GPs that men consult may increase the likelihood of prostate cancer being raised for discussion by men or GPs initiating discussion on it, or it may result in a less personalised relationship and avoidance of discussion of the topic.

The issue of over-treatment of indolent disease fuels the debate between GPs actively engaging in screening of men for prostate cancer versus relying on patients to initiate the conversation. While over-treating low-risk patients has been an identified issue, active surveillance has been an increasingly preferred option for men diagnosed with low-risk prostate cancer [18]. Active surveillance is a conservative management technique of periodically observing PSA levels and biopsies, to monitor disease progression of low-risk neoplasms [25]. This technique prevents unnecessary treatment and treatment-related morbidities of prostate cancer with no metastatic potential [26]. Reclassification of cancer categories was introduced in 2013, in part to address the issue of over-treatment. Cancer is now morphologically classified as a grade from 1 to 5 instead of on a range from 6 to 10 [27]. This new system provides a less confronting number range as well as improved prognostic accuracy, which may encourage GPs to engage in a conversation about testing, and reduce fears and uncertainty in discussing management options when test results are received.

The strength of this study lies in the design, which used a qualitative approach to learn about the perspectives of men without prostate cancer. Qualitative research makes no claim to generalisability in the positivist sense; [6] applicability is assessed by whether results can be validly applied in other contexts [6]. When dealing with meaning, it cannot be assumed that people who share certain characteristics will share meanings. Identical events can be understood in contrasting or subtly different ways.

Limitations of the research should be acknowledged. There was potentially a pre-determined set of characteristics of the type of man who volunteered for a research study of perspectives on prostate cancer. The participants may have increased awareness and may not necessarily reflect the 'average man'. It is however, difficult to envision an alternate method of recruitment. The participant sample that ensued did consist of a high proportion of men from Men’s Sheds and Rotary Clubs who were active advocates of prostate cancer prevention and had a better understanding on the topic than other men. The characteristics of these participants included being from a higher socioeconomic group as well as being naturally open to discussion on health-related issues affecting men their age. Many of these men had knowledge of prostate cancer from the numerous men’s health events held in their club [28]. As such, the sample of participants recruited in this study had an unusually large number of men who had a better understanding of prostate cancer than one would expect of a regular Australian male. However, these men acted as ‘key informants’ to the topic and provided valuable insight into observed perspectives and attitudes from their peers around them. The overall participant sample also included men who had a lesser understanding of prostate cancer as well and including both types of participants allowed a clear contrast in behavioural attributes relating to prostate care. Also, there were no Aboriginal participants in this study. The inclusion of this population could have possibly revealed unexplored perspectives of men on prostate health. The lack of homosexual male volunteers also limited insights into specific experiences and challenges faced by these men.

## Conclusions

Both prospects of over- and under-diagnosis pose a conundrum on the appropriate means of approaching prostate health. As opposed to encouraging or discouraging prostate checks, a focus on informed decision-making is required. With the current dependence on case-finding of prostate cancer, results of this study identifying factors preventing men from addressing prostate health, and existing literature documenting challenges of inaccessibility to care and poorer outcomes upon diagnosis for men in regional areas, [29] a systematic approach is required to improve prognosis for the future generation of Australian men.

## Supporting information

S1 Fig Undiagnosed men semi-structured interview protocol. (DOCX)

S1 Table Consolidated criteria for reporting qualitative studies (COREQ): 32-item checklist. (DOCX)
