# Supplementary figures and images for: Prostate cancer awareness, case-finding, and early diagnosis: Interviews with undiagnosed men in Australia
Source: PLoS One. 2019 Mar 7;14(3):e0211539. doi: 10.1371/journal.pone.0211539 (PMC6405086; doi:10.1371/journal.pone.0211539)

S1 Fig


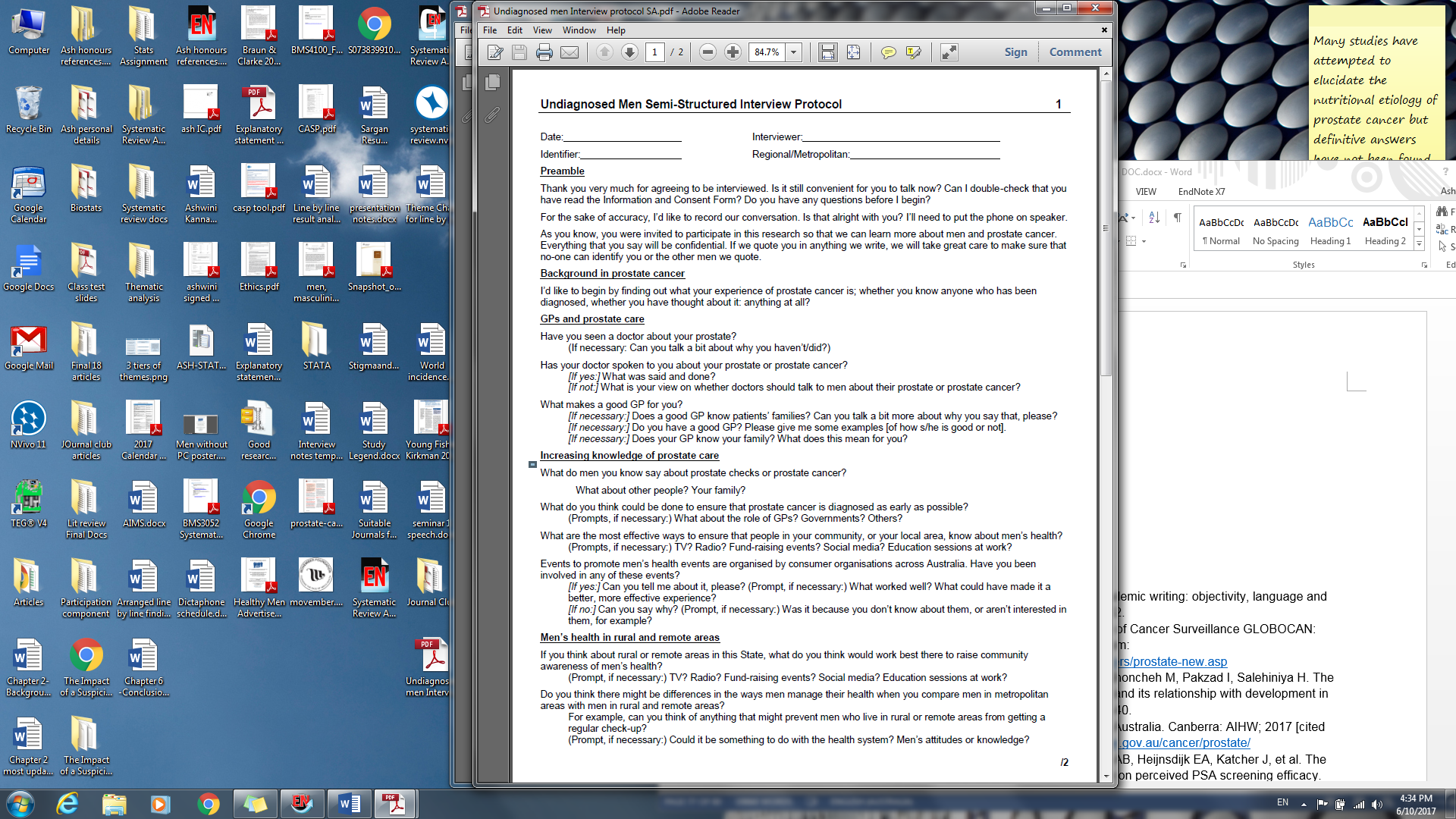

Supplement: S1 Fig — (DOCX) [file pone.0211539.s001.docx]
